# The *MAPT* H1 Haplotype Is a Risk Factor for Alzheimer’s Disease in *APOE* ε4 Non-carriers

**DOI:** 10.3389/fnagi.2019.00327

**Published:** 2019-12-04

**Authors:** Pascual Sánchez-Juan, Sonia Moreno, Itziar de Rojas, Isabel Hernández, Sergi Valero, Montse Alegret, Laura Montrreal, Pablo García González, Carmen Lage, Sara López-García, Eloy Rodrííguez-Rodríguez, Adelina Orellana, Lluís Tárraga, Mercè Boada, Agustín Ruiz

**Affiliations:** ^1^Center for Networked Biomedical Research on Neurodegenerative Diseases, National Institute of Health Carlos III, Ministry of Economy and Competitiveness, Madrid, Spain; ^2^Service of Neurology, University Hospital Marqués de Valdecilla, IDIVAL, University of Cantabria, Santander, Spain; ^3^Research Center and Memory Clinic, Fundació ACE, Institut Català de Neurociències Aplicades, Universitat Internacional de Catalunya, Barcelona, Spain

**Keywords:** Alzheimer’s disease, *MAPT*, H1H2, *APOE*, genetic association

## Abstract

An ancestral inversion of 900 kb on chromosome 17q21, which includes the microtubule-associated protein tau (*MAPT*) gene, defines two haplotype clades in Caucasians (H1 and H2). The H1 haplotype has been linked inconsistently with AD. In a previous study, we showed that an SNP tagging this haplotype (rs1800547) was associated with AD risk in a large population from the Dementia Genetics Spanish Consortium (DEGESCO) including 4435 cases and 6147 controls. The association was mainly driven by individuals that were non-carriers of the *APOE* ε4 allele. Our aim was to replicate our previous findings in an independent sample of 4124 AD cases and 3290 controls from Spain (GR@ACE project) and to analyze the effect of the H1 sub-haplotype structure on the risk of AD. The H1 haplotype was associated with AD risk (OR = 1.12; *p* = 0.0025). Stratification analysis showed that this association was mainly driven by the *APOE* ε4 non-carriers (OR = 1.15; *p* = 0.0022). Pooled analysis of both Spanish datasets (*n* = 17,996) showed that the highest AD risk related to the *MAPT* H1/H2 haplotype was in those individuals that were the oldest [third tertile (>77 years)] and did not carry *APOE* ε4 allele (*p* = 0.001). We did not find a significant association between H1 sub-haplotypes and AD. H1c was nominally associated but lost statistical significance after adjusting by population sub-structure. Our results are consistent with the hypothesis that genetic variants linked to the *MAPT* H1/H2 are tracking a genuine risk allele for AD. The fact that this association is stronger in *APOE* ε4 non-carriers partially explains previous controversial results and might be related to a slower alternative causal pathway less dependent on brain amyloid load.

## Introduction

Dementia is related to many underlying pathologies, with Alzheimer’s disease (AD) being the most common. AD is pathologically defined by the deposits of two proteins: tau which accumulates intracellularly and β-amyloid that accumulates extracellularly and within the walls of the blood vessels of the central nervous system. Dementia of AD-type is a complex entity with a common clinical syndrome that is likely to be reached by different routes influenced by genetic and environmental factors. This complexity is likely to have contributed to the constant failures of AD clinical trials. In particular, therapies based on the amyloid cascade hypothesis have not demonstrated any disease-modifying effect, despite some of these attempts have been proved to be effective in permanently removing brain β-amyloid plaques (Nicoll et al., 2019). This has led the pharmaceutical industry to focus on other therapeutic targets such as the tau protein (Corvol and Buée, 2019).

Neurofibrillary tangles composed of truncated and hyperphosphorylated tau proteins are hallmarks of AD pathology (Goedert, 2004). Tau protein plays an essential role in the central nervous system by promoting microtubule assembly and stability in neuronal cells. Emerging evidence supports that tau function is essential for normal synaptic mechanisms and it may be dysregulated in AD potentially through interaction with genetic risk factors in an Aβ-dependent or Aβ-independent manner (Dourlen et al., 2019). Additionally, tau has been proposed to spread through the brain from neuron to neuron by a “prion-like” mechanism (Clavaguera et al., 2015).

Tau protein is encoded by the *MAPT* gene (*MAPT*: OMIM: ^∗^157140), located at chromosome 17q21-22. There are two common *MAPT* extended haplotypes in Caucasians resulting from an ancestral inversion: H1 and H2. The H1 haplotype has been linked with familial and sporadic neurodegenerative disorders like progressive supranuclear palsy (PSP) (Conrad et al., 1997; Baker et al., 1999; Higgins et al., 2000; Pastor et al., 2004, 2016), corticobasal degeneration (CBD) (Houlden et al., 2001), Frontotemporal Dementia (FTD) (Verpillat et al., 2002), and Parkinson’s disease (PD) (Martin et al., 2001; Simón-Sánchez et al., 2009; Pastor et al., 2016). Genome-wide association studies (GWAS) have shown that the *MAPT* H1 haplotype is associated with CBD, PSP, and FTD (Yokoyama et al., 2017).

The H1 haplotype is further divided into sub-haplotypes, of those H1c has been associated with several neurodegenerative diseases (Myers et al., 2005; Heckman et al., 2019). H1c has been associated with higher levels of tau in plasma and CSF (Myers et al., 2007; Chen et al., 2017) and inconsistently with AD (Myers et al., 2005).

Although recent genetic studies show that several AD GWAS-associated genes, especially BIN1, are potentially involved in tau pathways (Dourlen et al., 2019), *MAPT* itself has not emerged until recently as a locus associated with AD. The IGAP consortium found a significant association between an SNP tagging *MAPT* H1 haplotype (rs2732703) and AD in subjects not carrying APOE ε4, however, the authors concluded that their conditional analysis pointed out that *MAPT* was probably not the causal gene (Jun et al., 2016).

Microtubule-associated protein tau H1/H2 haplotype frequency varies according to populations, with H2 frequency being maximum in the Mediterranean region and decreasing gradually as we move away from that area (Donnelly et al., 2010). These differences might contribute to explain controversial results among studies, as genetic stratification in genetically heterogeneous populations might constitute an important confounder. It is therefore essential to study these variants in large genetically homogeneous populations. In a previous study, we showed that H1 *MAPT* haplotype was strongly associated with risk of PSP, PD, and AD in 4435 cases and 6147 controls from Spain (Pastor et al., 2016). Therefore, it seems that *MAPT* H1/H2 haplotypes might play a relevant role within the genetic architecture of several neurodegenerative pathologies in our country. It is worth mention that the prevalence of the haplotype H2 in our control sample was one of the highest reported worldwide (29%) (Pastor et al., 2016).

In the present study, we aimed to replicate our previous findings in an independent sample. To do that, we assessed the association between the AD risk and the *MAPT* H1/H2 haplotype and H1 sub-haplotypes in 4,124 AD cases and 3,290 controls from Spain (GR@ACE/DEGESCO project).

## Materials and Methods

A detailed description of the methods and population of the GR@ACE study has been published elsewhere (Moreno-Grau et al., 2019)^1^.

### Population

The GR@ACE study comprises 4,120 AD cases and 3,289 control individuals. Cases were recruited from Fundació ACE, Institut Català de Neurociències Aplicades (Barcelona, Spain). Diagnoses were established by a neurology working-group according to the DSM-IV criteria for dementia and to the National Institute on Aging and Alzheimer’s Association’s (NIA-AA) 2011 guidelines for defining AD. In the present study, we considered AD cases, dementia individuals diagnosed with probable or possible AD at any moment of their clinical course.

Control individuals were recruited from three centers: Fundació ACE (Barcelona, Spain), Valme University Hospital (Seville, Spain) and the Spanish National DNA Bank Carlos III (University of Salamanca, Spain)^2^. Written informed consent was obtained from all participants. The Ethics and Scientific Committees have approved this research protocol (Acta 25/2016, Ethics Committee, Hospital Clinic I Provincial de Barcelona, Barcelona, Spain).

### Genotyping, Quality Control, Imputation, and Statistical Analysis

Participants were genotyped using the Axiom 815K Spanish Biobank array (Thermo Fisher). Genotyping was performed in the Spanish National Center for Genotyping (CeGEN, Santiago de Compostela, Spain).

We removed samples with genotype call rates below 97%, excess heterozygosity, duplicates, samples genetically related to other individuals in the cohort or sample mix-up (PIHAT > 0.1875). If a sex discrepancy was detected, the sample was removed unless the discrepancy was safely resolved. To detect population outliers of non-European ancestry (>6 SD from European population mean), principal component analysis (PCA) was conducted using SMARTPCA from EIGENSOFT 6.1.4.

We removed variants with a call rate < 95% or that grossly deviated from Hardy–Weinberg equilibrium in controls (*P*-value ≤ 1 × 10^–4^), markers with a different missing rate between case and control (*P*-value < 5 × 10^–4^ for the difference) or minor allele frequency (MAF) below 0.01. Imputation was carried out using Haplotype reference consortium (HRC) panel in Michigan Imputation servers^3^. Only common markers (MAF > 0.01) with a high imputation quality (*R*^2^ > 0.30) were selected to conduct downstream association analyses.

### Statistical Analysis

Allelic and genotypic frequencies were compared using χ^2^ statistics. Adjusted analyses were performed using multiple logistic regression. We used rs1800547 to tag *MAPT* H2 haplotype. Additionally, we used six tagging variants (rs1467967, rs242557, rs3785883, rs2471738, rs8070723, rs7521), to construct most common *MAPT* H1 sub-haplotypes as previously described (Pittman et al., 2005; Allen et al., 2014). To control for population sub-structure, results were co-variated by the main four principal components detected in this population (Moreno-Grau et al., 2019). All analyses were performed in PLINK 1.7.

## Results

We included 3290 controls with a mean age of 54.3 ± 14.4 years, and 48.9% of females, and 4124 AD cases with a mean age of 79.0 ± 7.5 years, 69.6% of females. No gross deviation from Hardy Weinberg equilibrium was found in controls for any of the *MAPT* studied variants (Table 1).

**TABLE 1:** Hardy–Weinberg equilibrium test in controls.

**SNP**	**Genotypes**	**Observed (HET)**	**Expected (HET)**	***P*-value**
rs1467967	264/1346/1679	0.41	0.41	0.83
rs242557	346/1426/1517	0.43	0.44	0.69
rs3785883	128/955/2206	0.29	0.30	0.06
rs2471738	89/991/2209	0.30	0.29	0.08
rs8070723	292/1328/1669	0.40	0.41	0.24
rs7521	614/1564/1111	0.48	0.49	0.13

Table 2 shows the allelic and genotypic frequency distribution of the SNPrs1800547 tagging the H1/H2 haplotype. We found a statistically significant overrepresentation of the *MAPT* H1 haplotype, present in 73.3% of AD compared to 71.1% of controls (*p* = 0.00025). When we stratified the sample by *APOE* ε4 status, the association of the H1 haplotype was driven by non-carriers of *APOE* ε4 (*p* = 0.0022) (Table 2). The association followed exactly the same pattern as our previous study (Pastor et al., 2016). Pooling both Spanish population confirmed that *MAPT* H1 was significantly more common in AD compared to controls (73.5 versus 70.7% respectively; *p* = 1.0 × 10^–5^), and this association was predominantly due to the *APOE* ε4 non-carriers (*p* = 8.0 × 10^–5^) (Table 2).

**TABLE 2:** Microtubule-associated protein tau H1/H2 haplotypes and AD risk.

	**Control (%)**	**AD (%)**	**Genotype *P*-value**	**Allelic *P*-value**	**Allelic OR (95%CI)**
**ALL**					
H2H2	290 (8.8)	328 (8.0)	*p* = 0.008	*p* = 0.00025	1.12 (1.04–1.20)
H1H2	1324 (40.2)	1546 (37.5)			
H1H1	1676 (50.9)	2250 (54.6)			
H1 frequency	0.711	0.733			
**APOE4+**					
H2H2	58 (8.3)	136 (8.3)	*p* = 0.42	*p* = 0.32	1.07 (0.93–1.23)
H1H2	286 (40.7)	625 (37.9)			
H1H1	358 (51.0)	887 (53.8)			
H1 frequency	0.714	0.728			
**APOE4−**					
H2H2	232 (9.0)	190 (7.7)	*p* = 0.009	*p* = 0.0022	1.15 (1.05–1.25)
H1H2	1033 (40.1)	913 (37.2)			
H1H1	1311 (50.9)	1353 (55.1)			
H1 frequency	0.709	0.737			
**ALL (Pastor et al., 2016)**					
H2H2	532 (9.15)	344 (8.34)	*p* = 0.001	*p* = 0.00051	1.12 (1.05–1.19)
H1H2	2444 (42.03)	1614 (39.11)			
H1H1	2839 (48.82)	2169 (52.56)			
H1 frequency	0.698	0.721			
**APOE4+ (Pastor et al., 2016)**					
H2H2	78 (9.07)	139 (8.50)	*p* = 0.88	*p* = 0.65	1.03 (0.91–1.18)
H1H2	345 (40.12)	655 (40.04)			
H1H1	437 (50.81)	842 (51.47)			
H1 frequency	0.709	0.715			
**APOE4− (Pastor et al., 2016)**					
H2H2	343 (8.46)	160 (8.16)	*p* = 0.001	*p* = 0.0025	1.14 (1.05–1.24)
H1H2	1701 (41.97)	730 (37.24)			
H1H1	2009 (49.57)	1070 (54.59)			
H1 frequency	0.706	0.732			
**ALL pooled**					
H2H2	822 (9.0)	672 (8.1)	*p* = 1.03 × 10^–5^	*p* = 1.0 × 10^–5^	1.126 (1.075–1.127)
H1H2	3769 (41.4)	3160 (38.3)			
H1H1	4515 (49.6)	4419 (53.6)			
H1 frequency	0.703	0.727			
**APOE4+ pooled**					
H2H2	136 (8.7)	275 (8.4)	*p* = 0.52	*p* = 0.286	1.053 (0.958–1.157)
H1H2	631 (40.4)	1280 (39.0)			
H1H1	795 (50.9)	1729 (52.6)			
H1 frequency	0.711	0.721			
**APOE4− pooled**					
H2H2	575 (8.7)	350 (7.9)	*p* = 5.16 × 10^–5^	*p* = 8.0 × 10^–5^	1.148 (1.08–1.22)
H1H2	2734 (41.2)	1643 (37.2)			
H1H1	3320 (50.1)	2423 (54.9)			
H1 frequency	0.707	0.735			

Table 3 shows the sub-haplotypes of *MAPT* in cases and controls. In addition to the protective effect of H2, only H1c was statistically significantly associated with AD. However, when we adjusted by the four main genetic components H1c was not statistically significant. After stratifying by *APOE* ε4 these results did not change substantially, and in addition to H2, only two rare sub-haplotypes (H1u and H1v) were nominally associated with AD. After adjustment, none of the associations survived multiple comparisons correction (Table 4).

**TABLE 3:** Microtubule-associated protein tau sub-haplotypes.

**Haplotype ID**	**Haplotype^∗^**	**Cases**	**Controls**	***P***	**Adjusted *P*-value^∗∗^**
H1o	AAACAA	0.022	0.021	0.63	0.47
H1y	AAATAG	0.013	0.011	0.31	0.22
H1d	AAGCAA	0.069	0.072	0.60	0.30
H1u	AAGCAG	0.025	0.022	0.19	0.14
H1q	AAGTAA	0.012	0.013	0.52	0.22
H1c	AAGTAG	0.114	0.104	0.05	0.14
H1h	AGACAA	0.050	0.047	0.51	0.25
H1l	AGACAG	0.046	0.044	0.65	0.44
H1t	AGATAG	0.011	0.010	0.64	0.30
H1e	AGGCAA	0.075	0.077	0.75	0.90
H1j	AGGCAG	0.016	0.016	0.78	0.95
H2	AGGCGG	0.283	0.309	0.0009	0.0008
H1z	GAATAG	0.014	0.011	0.12	0.07
H1i	GAGCAA	0.038	0.040	0.69	0.72
H1m	GAGCAG	0.026	0.023	0.32	0.36
H1f	GGACAA	0.014	0.014	0.95	0.75
H1v	GGATAG	0.014	0.012	0.27	0.08
H1b	GGGCAA	0.159	0.156	0.60	0.31

*^∗^rs1467967; rs242557; rs3785883; rs2471738; rs8070723; rs7521; ^∗∗^adjusted by the four genetic principal components.*

**TABLE 4:** Microtubule-associated protein tau sub-haplotypes stratified by APOE ε4 status.

**Haplotype ID**	**Haplotype^∗^**	**OR**	***P*-value**	**Adjusted *P*-value^∗∗^**
**APOE4+**				
H1o	AAACAA	1.17	0.61	0.63
H1f	GGACAA	1.55	0.31	0.20
H1h	AGACAA	0.96	0.81	0.93
H1i	GAGCAA	1.04	0.83	0.90
H1d	AAGCAA	0.85	0.26	0.18
H1b	GGGCAA	1.08	0.43	0.37
H1e	AGGCAA	1.02	0.88	0.93
H2	AGGCGG	0.93	0.28	0.23
H1z	GAATAG	1.77	0.14	0.10
H1v	GGATAG	0.95	0.86	0.92
H1t	AGATAG	1.46	0.37	0.28
H1c	AAGTAG	1.13	0.27	0.37
H1l	AGACAG	1.01	0.95	0.64
H1m	GAGCAG	1.07	0.78	0.89
H1u	AAGCAG	0.91	0.71	0.76
H1j	AGGCAG	0.66	0.18	0.21
**APOE4−**				
H1q	AAGTAA	1.12	0.56	0.86
H1g	GAACAA	0.94	0.81	0.92
H1o	AAACAA	1.12	0.46	0.40
H1f	GGACAA	0.93	0.74	0.93
H1h	AGACAA	1.11	0.34	0.19
H1i	GAGCAA	0.89	0.37	0.46
H1d	AAGCAA	1.02	0.83	0.83
H1b	GGGCAA	1.04	0.55	0.34
H1e	AGGCAA	0.94	0.46	0.90
H2	AGGCGG	0.88	0.00	0.00083
H1z	GAATAG	1.33	0.20	0.24
H1y	AAATAG	1.30	0.25	0.35
H1v	GGATAG	1.41	0.13	0.04
ND	AGATAG	1.03	0.91	0.65
H1c	AAGTAG	1.12	0.10	0.37
H1l	AGACAG	1.06	0.56	0.60
H1m	GAGCAG	1.21	0.20	0.33
H1u	AAGCAG	1.41	0.03	0.04
H1j	AGGCAG	1.15	0.46	0.30

*^∗^rs1467967; rs242557; rs3785883; rs2471738; rs8070723; rs7521; ^∗∗^adjusted by the four genetic principal components.*

**FIGURE 1:**
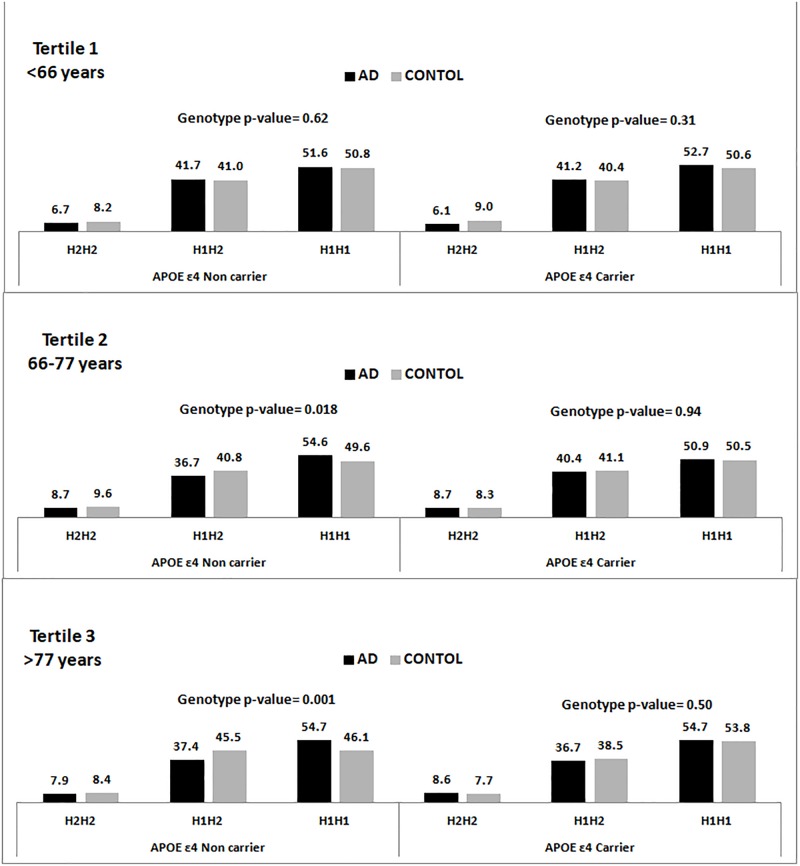
Genotype frequency distribution of the rs1800547 SNP tagging *MAPT* H1/H2 haplotype stratified by *APOE* ε4 status and age tertiles.

Figure 1 shows the genotypic distribution of the SNP rs1800547 (*MAPT* H1/H2) stratified by *APOE* ε4 across age tertiles for the pooled population. In the available entire sample of 15,522 individuals, we appreciated that within the APOE ε4 non-carriers the association between *MAPT* H1/H2 and AD increased with age, and it was stronger in the oldest individuals.

## Discussion

Our data, from a large and homogeneous single country population, shows that individuals carrying the H1 *MAPT* haplotype are at higher risk to develop AD dementia. The association is predominantly present in the *APOE* ε4 non-carriers and it is stronger in the eldest. These results replicate our previous findings (Pastor et al., 2016) and are concordant with the IGAP study (Jun et al., 2016) showing an association of another SNP tagging H1 (rs2732703) with AD only in the *APOE* ε4 non-carriers.

Our pooled analysis, including 15522 individuals, strongly support an etiological role of the *MAPT* region in AD. This association has been difficult to replicate, and results of previous studies assessing *MAPT* H1/H2 haplotype as a risk factor for AD have been controversial, although some of them were considerably under-powered (Russ et al., 2001; Mukherjee et al., 2007; Babić Leko et al., 2018). A robust statistical association only emerged in large sample studies and meta-GWAS, after stratifying by *APOE* ε4 (Jun et al., 2016; Pastor et al., 2016). An alternative interpretation of our results would be that the causal variant could be in linkage disequilibrium with the *MAPT* H1 haplotype but outside the *MAPT* gene, as other authors have suggested (Jun et al., 2016). A less likely explanation might be contamination of non-AD tauopathies in *APOE* ε4 non-carriers.

There are several factors that might be related to our findings. Taken together, our two studies, comprise one of the largest single-country population assessing *MAPT* H1/H2 and AD risk to date. This is of special value, as the inversion haplotype frequency has been shown to differ significantly across populations, and it is estimated to be 20% in Europeans, 6% in Africans, and less than 1% in East Asians (Holzer et al., 2004). This ethnic variability might cause population sub-structure biasing the results in countries with a high degree of population admixture. This is likely to be less problematic in our study as our sample come from a single country, and we have tested population sub-structure in Spain which does not represent a substantial problem for common genetic variants analyses (Moreno-Grau et al., 2019). Additionally, in our sub-haplotype analysis, we controlled for population sub-structure by adjusting for genetic principal components. It is worth mentioning that, despite it is commonly reported that the inversion is found at a frequency of around 20% throughout Europe, it shows a great range of frequencies within Europe (from 5 to 37.5%). The H2 haplotype, which identifies the inversion, is most frequent around the Mediterranean decreasing outward in all directions (Donnelly et al., 2010). Our controls presented the inversion in nearly 30% of individuals, this high frequency of the H2 haplotype has increased our power to detect the association compared to other populations in which this variant is less prevalent.

A potential limitation of our study is the age difference between the cases and the controls, which are significantly younger. However, this could only jeopardize the validity of our findings in the case of a survival bias. But to our knowledge, the MAPT H1/H2 haplotype has not been associated with mortality. The most likely consequence of our age unbalance is a decrease in our power to detect the association, as some of the controls carrying the H1 haplotype may still develop AD in the future. It is therefore likely that we are underestimating the true association between the H1/H2 haplotype and AD.

Microtubule-associated protein tau H1 has been associated with many neurodegenerative diseases: PSP, PD, CBD, FTD, and AD. It has been reported that the *MAPT* H1 is more efficient at driving gene expression than the H2 haplotype (Kwok et al., 2004). This has been shown to be particularly true with the H1c sub-haplotype (Myers et al., 2007). However, the H1 sub-haplotype association with AD is controversial, and H1c findings have been difficult to replicate (Myers et al., 2005; Abraham et al., 2009; Allen et al., 2014). It is likely that population stratification might have played a role. In our data H1c was nominally associated with AD, however, the statistical significance was lost after adjusting by the principal components supporting the notion that no H1sub-haplotype is specifically increasing AD-risk, and stratification by APOE ε4 did not change substantially these results.

Our results add further evidence for an etiological role of *MAPT* gene variants in clinical AD, supporting the role of the *APOE* ε4 allele as a modulator of this association. As seen in our previous study in population from Spain (Pastor et al., 2016) and by the international consortium IGAP (Jun et al., 2016), this association is significantly stronger in APOE ε4 non-carriers. Recent studies with tau and amyloid PET supports a view of AD as a tauopathy driven by amyloid, suggesting that tau pathology would appear in middle temporal lobe earlier than amyloid deposits, but the co-occurrence of both would be needed for tau pathology to expand beyond the temporal lobes (Schöll et al., 2016). This is also coherent with the new findings of AD meta-GWAS which shows the involvement of both amyloid and tau pathways (Dourlen et al., 2019; Kunkle et al., 2019). Therefore, it seems that tau and amyloid deposits might follow, at least initially, independent trajectories up to the point when they reach a threshold in which β-amyloid might accelerate tau pathology. A recent single case publication showing that a patient with a presenilin 1 mutation was resistant to cognitive impairment, likely due to a homozygous mutation in *APOE*, supports the hypothesis that *APOE* might play an important role in this tau pathology acceleration (Arboleda-Velasquez et al., 2019). On the other hand, *APOE* status is associated to prevalence of brain amyloid pathology, as shown by a large PET and CSF study in non-demented population that found that *APOE* ε4 carriers had two to three times higher prevalence than non-carriers (Jansen et al., 2015).

We hypothesize that the *MAPT* H1 variant might increase the risk of tau pathology which might be related to different amyloid thresholds to disparate tau pathology. We speculate that the association is only significant in the *APOE* ε4 non-carriers because in the carriers the amyloid would mask the H1 *MAPT* effect, while in the non-carriers the “tau etiologic pathway” would play a more relevant role and might be able to increase AD risk with a lower amyloid involvement. It is likely that this phenomenon might take longer to develop which might explain why this association is not significant in the youngest patients and stronger in the third tertile. This hypothesis could be tested studying the trajectory of individuals classified according to *APOE* ε4 status and *MAPT* H1/H2 haplotype in prospective cohorts with sequential amyloid and tau PET assessments.

Our study highlights the complexity of AD and suggests the existence of different pathogenic routes influenced by the genetic background. To look for successful therapeutic strategies it will be very important to take into account this mechanistic diversity and to combat specifically the pathology demonstrated on each afflicted individual.

## Member of the GR@ACE Study Group

Research Center and Memory Clinic, Fundació ACE, Institut Català de Neurociències Aplicades, Universitat Internacional de Catalunya, Barcelona, Spain: C. Abdelnour, N. Aguilera, E. Alarcon, M. Alegret, M. Boada, M. Buendia, P. Cañabate, I. de Rojas, S. Diego, A. Espinosa, A. Gailhajenet, P. García González, S. Gil, M. Guitart, I. Hernández, M. Ibarria, A. Lafuente, E. Martín, A. Mauleón, G. Monté-Rubio, L. Montrreal, S. Moreno-Grau, M. Moreno, A. Orellana, G. Ortega, A. Pancho, E. Pelejà, A. Pérez-Cordon, S. Preckler, O. Rodríguez-Gómez, M. Rosende-Roca, A. Ruiz, S. Ruiz, A. Sanabria, M. A. Santos-Santos, O. Sotolongo-Grau, L. Tárraga, S. Valero, and L. Vargas. Center for Networked Biomedical Research on Neurodegenerative Diseases, National Institute of Health Carlos III, Ministry of Economy and Competitiveness, Madrid, Spain: C. Abdelnour, M. Alegret, M. Boada, P. Cañabate, A. Espinosa, I. Hernández, S. Moreno-Grau, G. Ortega, O. Rodríguez-Gómez, A. Ruiz, S. Ruiz, A. Sanabria, L. Tárraga, and S. Valero. Department of Surgery, Biochemistry and Molecular Biology, School of Medicine, University of Málaga, Málaga, Spain: E. Alarcon, I. Quintela, and L. M. Real. Grupo de Medicina Xenómica, Centro Nacional de Genotipado (CEGEN-PRB3-ISCIII), Universidade de Santiago de Compostela, Santiago, Spain: A. Carracedo and O. Maroñas. Fundación Pública Galega de Medicina Xenómica, CIBERER, IDIS, Santiago, Spain: A. Carracedo. Centro de Investigación Biomédica en Red de Diabetes y Enfermedades Metabólicas Asociadas, Hospital Clínico San Carlos, Madrid, Spain: A. Corbatón, M. T. Martínez, and M. Serrano-Rios. Centro Andaluz de Estudios Bioinformáticos, Seville, Spain: A. González Pérez and M. E. Sáez. Unidad Clínica de Enfermedades Infecciosas y Microbiología, Hospital Universitario de Valme, Seville, Spain: J. Macias, J. A. Pineda, and L. M. Real.

## Member of the DEGESCO Consortium

Unidad de Trastornos del Movimiento, Servicio de Neurología y Neurofisiología, Instituto de Biomedicina de Sevilla, Hospital Universitario Virgen del Rocío/CSIC/Universidad de Sevilla, Seville, Spain: A. D. Adarmes-Gómez, D. Buiza-Rueda, F. Carrillo, M. Carrión-Claro, P. Gómez-Garre, S. Jesús, M. A. Labrador Espinosa, D. Macias, P. Mir, and T. Periñán-Tocino. Network Center for Biomedical Research in Neurodegenerative Diseases, National Institute of Health Carlos III, Madrid, Spain: A. D. Adarmes-Gómez, R. Blesa, M. Boada, D. Buiza-Rueda, M. J. Bullido, M. Calero, F. Carrillo, M. Carrión-Claro, J. Clarimón, J. Fortea, A. Frank-García, P. Gómez-Garre, I. Hernández, S. Jesús, M. A. Labrador Espinosa, C. Lage, A. Lleó, S. López-García, D. Macias, A. Martín Montes, M. Medina, P. Mir, S. Moreno-Grau, J. Pérez Tur, T. Periñán-Tocino, G. Piñol Ripoll, A. Rábano, E. Rodríguez-Rodríguez, A. Ruiz, P. Sánchez-Juan, I. Sastre, L. Tárraga, and S. Valero. Research Center and Memory Clinic, Fundació ACE, Institut Català de Neurociències Aplicades, Universitat Internacional de Catalunya, Barcelona, Spain: E. Alarcón-Martín, M. Boada, I. de Rojas, I. Hernández, M. Marquié, G. Monté-Rubio, L. Montrreal, S. Moreno-Grau, A. Orellana, A. Ruiz, O. Sotolongo-Grau, L. Tárraga, and S. Valero. Department of Surgery, Biochemistry and Molecular Biology, School of Medicine, University of Málaga, Málaga, Spain: E. Alarcón-Martín, J. M. Cruz-Gamero, and J. L. Royo. Fundació per la Recerca Biomèdica i Social Mútua Terrassa, and Memory Disorders Unit, Department of Neurology, Hospital Universitari Mutua de Terrassa, School of Medicine, University of Barcelona, Barcelona, Spain: I. Álvarez, M. Diez-Fairen, and P. Pastor. Laboratorio de Genética, Hospital Universitario Central de Asturias, Oviedo, Spain: V. Álvarez. Instituto de Investigación Biosanitaria del Principado de Asturias, Oviedo, Spain: V. Álvarez, C. Martínez, and M. Menéndez-González. Department of Neurology, Hospital Universitario Son Espases, Palma, Spain: G. Amer-Ferrer. Unidad de Demencias, Hospital Clínico Universitario Virgen de la Arrixaca, Murcia, Spain: M. Antequera, C. Antúnez, A. Legaz, S. Manzanares, J. Marín-Muñoz, B. Martínez, V. Martínez, M. P. Vicente, and L. Vivancos. Servei de Neurologia, Hospital Universitari i Politècnic La Fe, Valencia, Spain: M. Baquero and J. A. Burguera. Unidad de Demencias, Servicio de Neurología y Neurofisiología, Instituto de Biomedicina de Sevilla, Hospital Universitario Virgen del Rocío/CSIC/Universidad de Sevilla, Seville, Spain: M. Bernal, E. Franco, M. Marín, and S. Rodrigo. Sant Pau Memory Unit, Neurology Department, Sant Pau Biomedical Research Institute, Hospital de la Santa Creu i Sant Pau, Universitat Autònoma de Barcelona, Barcelona, Spain: R. Blesa, J. Clarimón, J. Fortea, and A. Lleó. Centro de Biologia Molecular Severo Ochoa (CSIC), Universidad Autonoma de Madrid, Madrid, Spain: M. J. Bullido, A. Martín Montes, and I. Sastre. Instituto de Investigacion Sanitaria “Hospital la Paz”, Madrid, Spain: M. J. Bullido, T. del Ser, A. Frank-García, and M. Medina. CIEN Foundation, Queen Sofia Foundation Alzheimer Center, Madrid, Spain: M. Calero, A. B. Pastor, and A. Rábano. Instituto de Salud Carlos III, Madrid, Spain: M. Calero, S. Garcia Madrona, and G. Garcia-Ribas. Hospital Universitario Ramón y Cajal, Madrid, Spain: M. J. Casajeros. BIOMICs, Centro de Investigación Lascaray, Universidad del País Vasco UPV/EHU, Leioa, Spain: M. M. de Pancorbo. Neurology Service, Hospital Universitario La Paz (UAM), Madrid, Spain: A. Frank-García and A. Martín Montes. Alzheimer Research Center and Memory Clinic, Andalusian Institute for Neuroscience, Málaga, Spain: J. M. García-Alberca, S. Hevilla, and T. Marín. Neurology Service, Marqués de Valdecilla University Hospital, IDIVAL, University of Cantabria, Santander, Spain: C. Lage, S. López-García, E. Rodríguez-Rodríguez, and P. Sánchez-Juan. Hospital Donostia de San Sebastían, Donostia, Spain: A. López de Munáin. Fundación para la Formación e Investigación Sanitarias de la Región de Murcia, Murcia, Spain: S. Manzanares. Servicio de Neurología, Hospital de Cabueñes, Gijón, Spain: C. Martínez. Centro de Investigación y Terapias Avanzadas, Fundación CITA-Alzheimer, Donostia, Spain: P. Martínez-Lage Álvarez. Navarrabiomed, Pamplona, Spain: M. Mendioroz Iriarte. Servicio de Neurología, Hospital Universitario Central de Asturias, Oviedo, Spain: M. Menéndez-González. Barcelonaβeta Brain Research Center, Fundació Pasqual Maragall, Barcelona, Spain: J. L. Molinuevo. Unitat de Genètica Molecular, Institut de Biomedicina de València, CSIC, Valencia, Spain: J. Pérez Tur. Unidad Mixta de Neurologia y Genètica. Instituto de Investigación Sanitaria La Fe, Valencia, Spain: J. Pérez Tur. Unitat Trastorns Cognitius, Hospital Universitari Santa Maria de Lleida, Institut de Recerca Biomédica de Lleida, Lleida, Spain: G: Piñol Ripoll. BT-CIEN, Madrid, Spain: A. Rábano. Hospital Universitario La Princesa, Madrid, Spain: D. Real de Asúa. Hospital Clínic de Barcelona, Barcelona, Spain: R. Sanchez del Valle Díaz.

## Data Availability Statement

The datasets generated for this study can be accessed from the https://ega-archive.org/studies/EGAS00001003424.

## Ethics Statement

The studies involving human participants were reviewed and approved by Acta 25/2016, Ethics Committee, Hospital Clinic I Provincial de Barcelona, Barcelona, Spain. The patients/participants provided their written informed consent to participate in this study.

## Author Contributions

PS-J and AR: data collection, data analysis, study design, manuscript drafting, and manuscript critical review. SM, IR, IH, SV, MA, LM, PG, CL, SL-G, ER-R, and AO: data collection, data analysis, and manuscript critical review. LT and MB: data collection, data analysis, manuscript critical review, and study design.

## Conflict of Interest

The authors declare that the research was conducted in the absence of any commercial or financial relationships that could be construed as a potential conflict of interest.

## Footnotes

1 https://www.biorxiv.org/content/10.1101/528901v1

2 www.bancoadn.org

3 https://imputationserver.sph.umich.edu
